# Association between pancreatic fibrosis and development of pancreoprivic diabetes after pancreaticoduodenectomy

**DOI:** 10.1038/s41598-021-02858-z

**Published:** 2021-12-07

**Authors:** Jung Min Lee, Hyung Sun Kim, Minyoung Lee, Ho Seon Park, Shinae Kang, Ji Hae Nahm, Joon Seong Park

**Affiliations:** 1 Pancreatobiliary Cancer Clinic, Department of Surgery, Gangnam Severance Hospital, Yonsei University College of Medicine, 20, Eonju-ro 63-gil, Gangnam-gu, Seoul, 06229 Republic of Korea; 2 Department of Internal Medicine, Yonsei University College of Medicine, Seoul, Korea; 3 Department of Internal Medicine, Severance Institute for Vascular and Metabolic Research, Gangnam Severance Hospital, Yonsei University College of Medicine, Seoul, Korea; 4 Department of Pathology, Gangnam Severance Hospital, Yonsei University College of Medicine, 20, Eonju-ro 63-gil, Gangnam-gu, Seoul, 06229 Republic of Korea

**Keywords:** Diseases, Endocrinology, Gastroenterology, Medical research, Oncology, Risk factors

## Abstract

This study investigated the correlation between pancreatic fibrosis (PF) and development of pancreoprivic diabetes after pancreaticoduodenectomy (PD). Ninety-five patients who underwent PD at Gangnam Severance Hospital between 2014 and 2017 were enrolled. PF grade was evaluated with alpha-smooth muscle actin (SMA) and Masson’s trichrome (TRC) staining. New-onset pancreoprivic diabetes and recurrence of disease were evaluated using fasting blood glucose measurement and radiography taken at 3-month intervals. Sixty-one patients did not have preoperative diabetes, however, 40 (65.6%) patients developed pancreoprivic diabetes after PD. High-grade PF was more common in the diabetes group than in the normal group (SMA, 42.5% vs. 28.6%, *P* = 0.747; TRC, 47.5% vs. 28.6%, *P* = 0.361). The 1-year cumulative incidence of hyperglycemia/pancreoprivic diabetes was higher with high-grade PF than low-grade PF (SMA, 94.4% vs. 73.0%, *P* = 0.027; TRC, 89.3% vs. 75.0%, *P* = 0.074). The SMA-TRC combined high-grade group had a higher proportion of primary pancreatic disease than the combined low-grade group (90.0% vs. 37.5%, *P* = 0.001). The 5-year disease-free survival of patients with pancreatic cancer was worse with high-grade PF than low-grade PF (SMA, 24.5% vs. 66.3%, *P* = 0.026; TRC, 23.6% vs. 58.4%, *P* = 0.047). In conclusion, patients with severe PF are more likely to develop pancreoprivic diabetes after PD and have worse disease-free survival.

## Introduction

Diabetes mellitus is classified as type 1 (β-cell destruction), type 2 (insulin resistance with secretory defect), and type 3 (other specific types) subtypes by the American Diabetes Association^1^. The term ‘pancreoprivic diabetes’, historically referred to as type 3c diabetes mellitus, is used to define diabetes caused by loss or destruction of the endocrine pancreas^2^. More than 75% of pancreoprivic diabetes result from chronic pancreatitis; however, this form of diabetes mellitus is also caused by cystic fibrosis, pancreatic cancer, and pancreatic resection^3^. Pancreoprivic diabetes develops in 12–20% of patients who undergo pancreaticoduodenectomy (PD)^4,5^. The loss of pancreatic parenchyma plays an important role in the pathogenesis of pancreoprivic diabetes^6^, which is characterized by insulin deficiency and increased peripheral insulin sensitivity^7^. The initial defects in insulin secretion are associated with increases in glucagon secretion that may contribute to early impairment of glucose tolerance^8^. The treatment of pancreoprivic diabetes is difficult and requires comprehensive management strategies, such as diet modification, exercise, oral medication, and insulin replacement therapy.

Pancreatic fibrosis (PF) destroys pancreatic tissue, which results in insulin deficiency^9^. PF may have various causes, such as alcohol abuse, pancreatitis, pancreatic duct obstruction, biliary disease, and other unknown etiologies^10^. Damage to one or all of the tissue compartments or cell types of the pancreas incites fibrosis, which leads to cell necrosis and/or apoptosis and the subsequent release of cytokines/growth factors^11^. Subsequently, the damaged cells are phagocytosed by macrophages, and the released cytokines activate resident fibroblast proliferation and transformation of these cells into myofibroblasts. PF secondary to pancreatitis or desmoplastic reactions is observed in patients undergoing PD. Moreover, the PD procedure itself induces tissue damage, which prompts progression of PF.

While PD has been associated with pancreoprivic diabetes and PF^12,13^, no studies have attempted to analyze the relationship between PF and pancreoprivic diabetes after PD. Therefore, we aimed to evaluate the effect of PF on the development of pancreoprivic diabetes after PD.

## Methods

### Patients

This study was approved by the institutional review board of the Gangnam Severance Hospital (IRB No. 3-2014-0024) with a waiver for informed consent. All experiments were performed in accordance with relevant guidelines and regulations.

**Figure 1:**
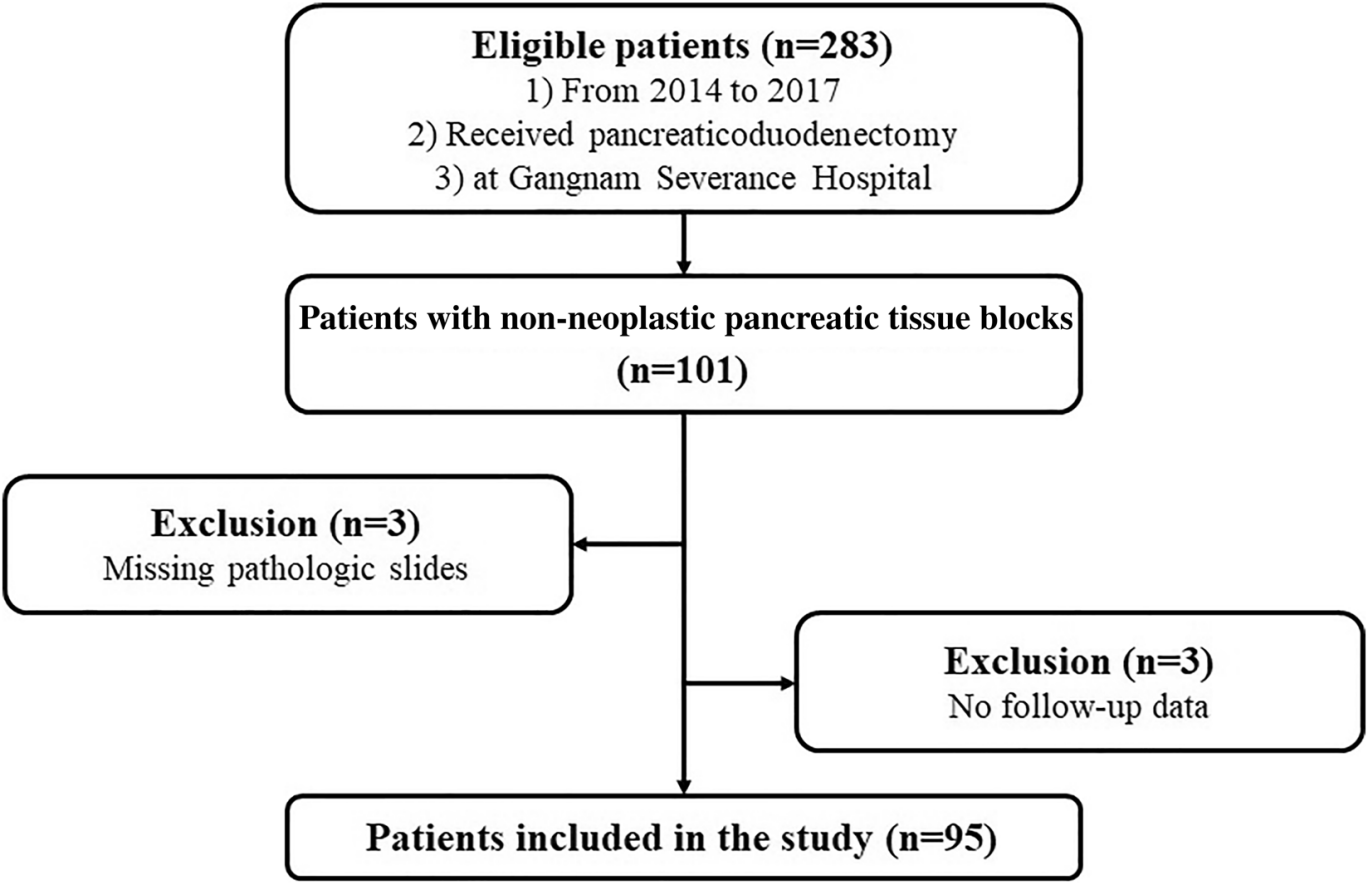
Patient flow chart.

A total of 283 patients underwent PD at the Gangnam Severance Hospital between 2014 and 2017. Representative non-neoplastic pancreatic tissue slides were reviewed and 101 patients with tissue blocks available for immunohistochemical staining were selected. Three patients with missing pathologic slides and another three patients with missing follow-up data were excluded. Consequently, 95 patients were included in this study (Fig. 1). The same PD technique was performed by experienced hepato-biliary-pancreatic surgeons in all patients. The patients in this study were diagnosed with a variety of benign and malignant conditions, including pancreatic ductal adenocarcinoma and biliary tract cancer.

### Clinicopathologic data

Baseline patient characteristics, such as age, sex, body mass index (BMI), American Society of Anesthesiologists score, previous operation history, and underlying disease, were investigated. Tumor markers, including carbohydrate antigen (CA) 19–9 and carcinoembryonic antigen (CEA), were measured before surgery. Perioperative clinical outcomes, including intraoperative and pathologic data, were examined. Intraoperative data included operation time and estimated blood loss. The pathologic findings were reported by a single pathologist. The duration of postoperative hospital stay, complications above Clavien-Dindo classification grade 3, and the number of patients who received adjuvant treatment were also recorded.

### Fibrosis grading of pancreatic resection margin

**Figure 2:**
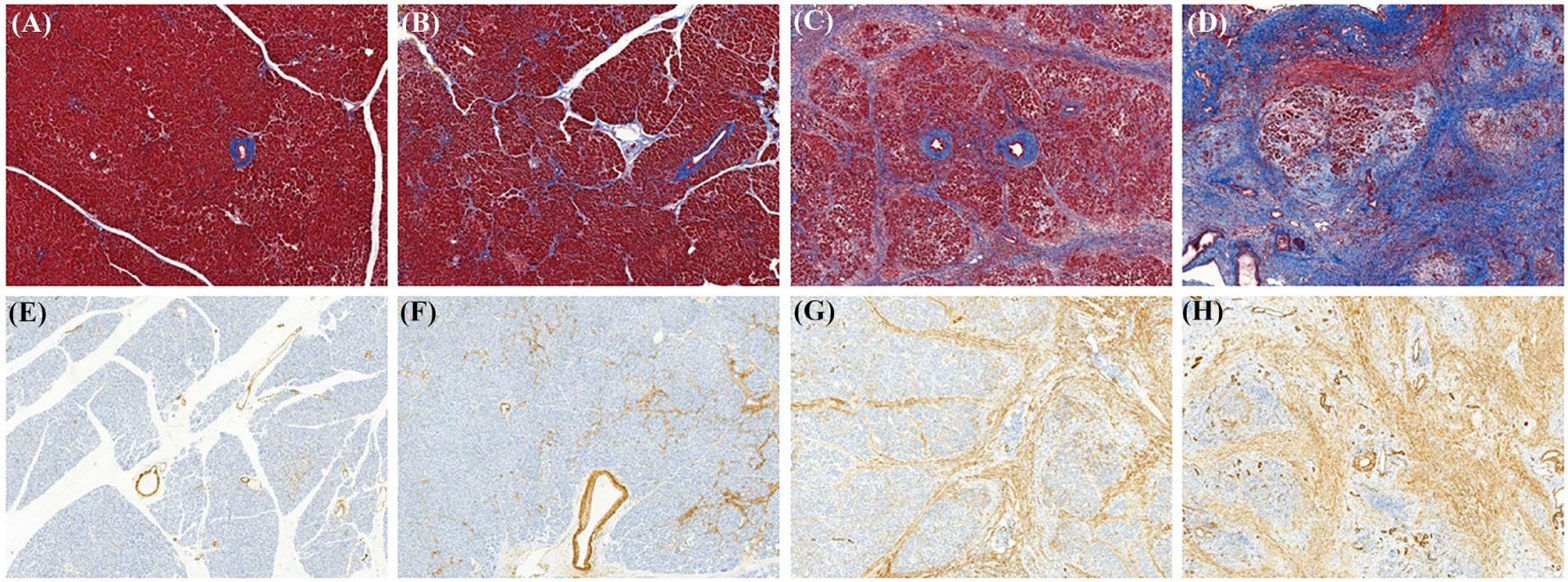
Histologic grade of pancreatic fibrosis (PF) and activated pancreatic stellate cells (PSC). PF was evaluated by Masson’s trichrome staining (TRC) and a four-grade scoring system; grade 0 (**A**), grade 1 (**B**), grade 2 (**C**), and grade 3 (**D**). PSC activity was evaluated by the immunoexpression of alpha-smooth muscle actin (SMA) and a four-grade scoring system; grade 0 (**E**), grade 1 (**F**), grade 2 (**G**), and grade 3 (**H**). (40X magnification).

Histopathologic and immunohistochemical analyses were performed on 95 non-neoplastic pancreatic specimens. Formalin-fixed paraffin-embedded tissues were sliced into serial 4-µm-thick sections that were stained with hematoxylin and eosin and Masson's trichrome (TRC). Immunohistochemical analysis was performed with anti-alpha-smooth muscle actin (α-SMA; Dako, Glostrup, Denmark). The tissue sections were deparaffinized, and endogenous peroxidase was blocked with 3% hydrogen peroxidase in a phosphate buffered saline solution. The specimens were incubated with the primary antibody at a 1:1000 dilution. The tissue sections were washed with phosphate buffered saline, and incubated with the biotinylated secondary antibody, followed by the streptavidin–peroxidase complex. The bound complex was visualized using diaminobenzidine liquid chromogen and counterstained using Mayer's hematoxylin. Fibrosis was graded with the four-stage scoring system described by Wellner et al*.*^14^, which included normal pancreatic parenchyma with no fibrotic changes (grade 0), mild fibrosis with thickening of periductal tissue (grade 1), moderate fibrosis with marked sclerosis of interlobular septa or intralobular sclerosis with no evidence of architectural changes (grade 2), and severe fibrosis with detection of architectural destruction or acinar cell atrophy (grade 3). To assess the grade of activated pancreatic stellate cells (PSC), a-SMA immunoexpression was evaluated. PSC were evaluated with the four-stage scoring system described by Watanabe et al*.*^15^, which included restriction of staining to periductal tissue (grade 0), weak positive staining that was irregular or multifocal (grade 1), weak positive staining that was consistently homogeneous or diffuse (grade 2), and strong positive staining that was always homogeneous and diffuse (grade 3) (Fig. 2).

### Postoperative onset of pancreoprivic diabetes and disease recurrence

Patients were seen at the outpatient clinic every 3 months to detect the onset of pancreoprivic diabetes or identify disease recurrence after surgery. Fasting blood glucose (FBG) levels were assessed at all visits. A FBG level between 110 and 126 mg/dl was considered as hyperglycemia. Patients with hyperglycemia were educated as to lifestyle modifications involving diet and exercise, but were not prescribed any medications. When the FBG levels exceeded 126 mg/dl, a glucose tolerance test was performed to confirm the diagnosis of pancreoprivic diabetes, for which medical treatment was initiated. The glucose tolerance test was performed using an oral glucose solution containing the equivalent of 75 g of anhydrous glucose dissolved in water as described by the World Health Organization^16^. Radiographic images and tumor markers were also evaluated at all visits. Disease recurrence was defined as positive findings on computed tomography images and elevation of tumor markers, such as CEA and CA19-9.

### Statistical analysis

All statistical calculations were made using SPSS Statistics version 25.0 (SPSS Inc., Chicago, IL, USA). Continuous variables were expressed as mean and standard deviation and were analyzed with Student’s t-test or the Mann–Whitney U test, as appropriate. Categorical variables were compared using the Chi-squared test or Fisher’s exact test. The cumulative incidence of hyperglycemia and disease-free survival of patients with pancreatic cancer were plotted using the Kaplan–Meier method, and intergroup differences in survival time were assessed with the log-rank test. Disease-free survival was defined as the interval between the dates of surgery and recurrence. Statistical significance was defined as *P* < 0.05.

## Results

### Demographics and clinicopathologic outcomes

Table 1 shows the baseline demographic and clinicopathologic outcomes of the patients in this study. The mean age of the patients was 64.2 years, and the study population included 60 males (63.2%). Among 34 participants who had diabetes mellitus, four (11.8%) and 19 (55.9%) patients were taking one type and more than one type of glucose-lowering medication, respectively (Supplementary table 1). Three (8.8%) patients were treated with insulin injection, and eight (23.5%) were suggested diet and life style modification for glycemic control. Primary pancreatic disease was diagnosed in 54 (56.8%) patients and included 36 (37.9%), 10 (10.5%), and eight (8.4%) cases of pancreatic ductal adenocarcinomas, intraductal papillary mucinous neoplasms (IPMNs), and benign pancreatic diseases, respectively. Non-pancreatic diseases were diagnosed in 41 patients (43.2%) and included 21 (22.1%) and 20 (21.1%) cases of cancers of the ampulla of Vater and common bile duct, respectively. After recovery from surgery, all patients with pancreatic ductal adenocarcinoma completed six cycles of gemcitabine-based adjuvant chemotherapy. Patients with malignancies in the ampulla of Vater or common bile duct, and who had stage II or higher cancer (n = 22), finished six cycles of 5-fluorouracil-based adjuvant chemotherapy. Nine (9.5%) patients received concurrent radiation therapy.

**Table 1:** Baseline demographics and clinicopathologic outcomes.

Variables	Total (N = 95)
Age (mean ± SD, year)	64.2 ± 11.7
Male gender	60 (63.2%)
BMI (mean ± SD, kg/m^2^)	23.4 ± 2.9
**ASA physical status**	
I	9 (9.5%)
II	41 (43.2%)
III	45 (47.3%)
Preoperative diabetes	34 (35.8%)
Previous operation history	24 (25.3%)
Pre-operative CA19-9 > 37U/mL	49 (51.6%)
Pre-operative CEA > 5 ng/mL	17 (17.9%)
**Pathology**	
Pancreatic origin	54 (56.8%)
Ductal adenocarcinoma	36 (37.9%)
IPMN	10 (10.5%)
Benign	8 (8.4%)
Non-pancreatic origin	41 (43.2%)
AOV cancer	21 (22.1%)
CBD cancer	20 (21.1%)
Operation time (mean ± SD, min)	461.9 ± 135.1
EBL (mean ± SD, mL)	1037.5.4 ± 653.3
Postoperative hospital stay (mean ± SD, day)	16.8 ± 7.5
Complication (CD grade ≥ 3a)	5 (5.3%)
Adjuvant treatment	56 (58.9%)

*SD* standard deviation, *BMI* body mass index, *ASA* American Society of Anesthesiologists, *CA19-9* carbohydrate antigen 19–9, *CEA* carcinoembryonic antigen, *IPMN* intraductal papillary mucinous neoplasm, *AOV* ampulla of Vater, *CBD* common bile duct, *EBL* estimated blood loss, *CD* Clavien–Dindo.

### TRC and SMA grade based on postoperative FBG

We excluded patients with preoperative diabetes when we analyzed the association between PF grade and FBG levels measured during the follow-up period. Sixty-one patients who did not have diabetes before surgery were classified into normal (normal FBG, n = 7), hyperglycemia (FBG of 110–126 mg/dL, n = 14), and pancreoprivic diabetes (FBG above 126 mg/dL, n = 40) groups (Table 2). Among 40 patients with pancreoprivic diabetes, 14 (35.0%) patients had 2-h plasma glucose levels above 200 mg/dl for the oral glucose tolerance test (Supplementary table 2). Grades 0–1 and 2–3 PF were classified as low- and high-grade PF, respectively. Our data demonstrated differences in the TRC and SMA grades among the three FBG level groups. The pancreoprivic diabetes group had more patients with high-grade TRC staining, compared to the other groups (normal, 28.6%; hyperglycemia, 28.6%; pancreoprivic diabetes, 47.5%; *P* = 0.361). In addition, the groups with higher FBG levels demonstrated higher SMA grades (normal, 28.6%; hyperglycemia, 35.7%; pancreoprivic diabetes, 42.5%; *P* = 0.747). The pancreoprivic diabetes group had more patients with high TRC and SMA grades compared to the other groups (normal, 28.6%; hyperglycemia, 28.6%; pancreoprivic diabetes, 35%; *P* = 0.229).

**Table 2:** Comparison of TRC and SMA grade by postoperative fasting blood glucose (FBG) levels.

	Total (N = 61)	Normal (N = 7, FBG ≤ 110)	Hyperglycemia (N = 14, 126 ≥ FBG > 110)	Pancreoprivic DM (N = 40, FBG > 126)	*P* value
**TRC**					0.361
Low (grade 0–1)	36 (59%)	5 (71.4%)	10 (71.4%)	21 (52..5%)	
High (grade 2–3)	25 (41%)	2 (28.6%)	4 (28.6%)	19 (47.5%)	
**SMA**					0.747
Low (grade 0–1)	37 (60.7%)	5 (71.4%)	9 (64.3%)	23 (57.5%)	
High (grade 2–3)	24 (39.3%)	2 (28.6%)	5 (35.7%)	17(42.5%)	
**TRC and SMA**					0.229
Combined low (grade 0–1)	32 (52.5%)	5 (71.4%)	9 (64.3%)	18 (45.0%)	
Combined high (grade 2–3)	20 (32.8%)	2 (28.6%)	4 (28.6%)	14 (35.0%)	
Low and high	4 (6.6%)	0 (0%)	1 (7.1%)	3 (7.5%)	
High and low	5 (12.5%)	0 (0%)	0 (0%)	5 (12.5%)	

*DM* diabetes mellitus, *TRC* trichrome, *SMA* smooth muscle actin.

The clinicopathologic differences between the TRC and SMA low- and high-grade groups were analyzed (Table 3). The combined low-grade group demonstrated higher BMI than the combined high-grade group (24.2 vs. 22.6, *P* = 0.029). There was a significant difference in the pathologic diagnosis between the low- and high-grade groups. Primary pancreatic diseases were much more common in the combined high-grade group than in the combined low-grade group (90% vs. 37.5%, *P* = 0.001). Non-pancreatic disease comprised only 10% of the conditions in the combined high-grade group.

**Table 3:** Clinicopathologic characteristics by fibrosis grade.

	Total (N = 52)	Combined low (N = 32)	Combined high (N = 20)	*P* value
Age (mean ± SD, year)	63.2 ± 12.1	61.5 ± 12.9	65.9 ± 10.4	0.180
Male gender	31 (59.6%)	21 (65.6%)	10 (50%)	0.264
BMI (mean ± SD, kg/m^2^)	23.7 ± 3	24.4 ± 3.1	22.6 ± 2.7	0.029
**ASA physical status**				0.579
I	5 (9.6%)	4 (12.5%)	1 (5%)	
II	30 (57.7%)	17 (53.1%)	13 (65%)	
III	17 (32.7%)	11 (34.4%)	6 (30%)	
Previous operation history	15 (28.8%)	10 (31.3%)	5 (25%)	0.872
Pre-op CA19-9 > 37U/mL	26 (50%)	13 (40.6%)	10 (65%)	0.087
Pre-op CEA > 5 ng/mL	6 (11.5%)	4 (12.5%)	2 (10%)	0.784
**Pathology**				0.001
Pancreatic origin	30 (57.7%)	12 (37.5%)	18 (90%)	
Ductal adenocarcinoma	19 (36.5%)	4 (12.5%)	15 (75%)	
IPMN	8 (15.4%)	6 (18.8%)	2 (10%)	
Benign	3 (5.8%)	2 (6.3%)	1 (5%)	
Non-pancreatic origin	22 (42.3%)	20 (62.5%)	2 (10%)	
AOV cancer	10 (19.2%)	8 (25%)	2 (10%)	
CBD cancer	12 (23.1%)	12 (37.5%)	0 (0%)	
R0 resection	48 (92.3%)	31 (96.9%)	17 (85%)	0.118
Operation time (mean ± SD, min)	465.2 ± 162.2	480.8 ± 197.7	440.3 ± 76.3	0.304
EBL (mean ± SD, mL)	1 108.9 ± 746.4	1 110 ± 807.8	1 107.0 ± 656.3	0.988
Postoperative hospital stay (day)	17.7 ± 8.9	19.6 ± 10.5	14.7 ± 4.2	0.053
Complication (CD grade ≥ 3a)	13 (25%)	3 (9.4%)	0 (0%)	0.276
Adjuvant treatment	29 (55.8%)	14 (43.8%)	15 (75%)	0.027

*SD* standard deviation, *BMI* body mass index, *ASA* American Society of Anesthesiologists, *CA19-9* carbohydrate antigen 19–9, *CEA* carcinoembryonic antigen, *IPMN* intraductal papillary mucinous neoplasm, *AOV* ampulla of Vater, *CBD* common bile duct, *EBL* estimated blood loss, *CD* Clavien–Dindo.

### Incidence of hyperglycemia and pancreoprivic diabetes by PF grade

**Figure 3:**
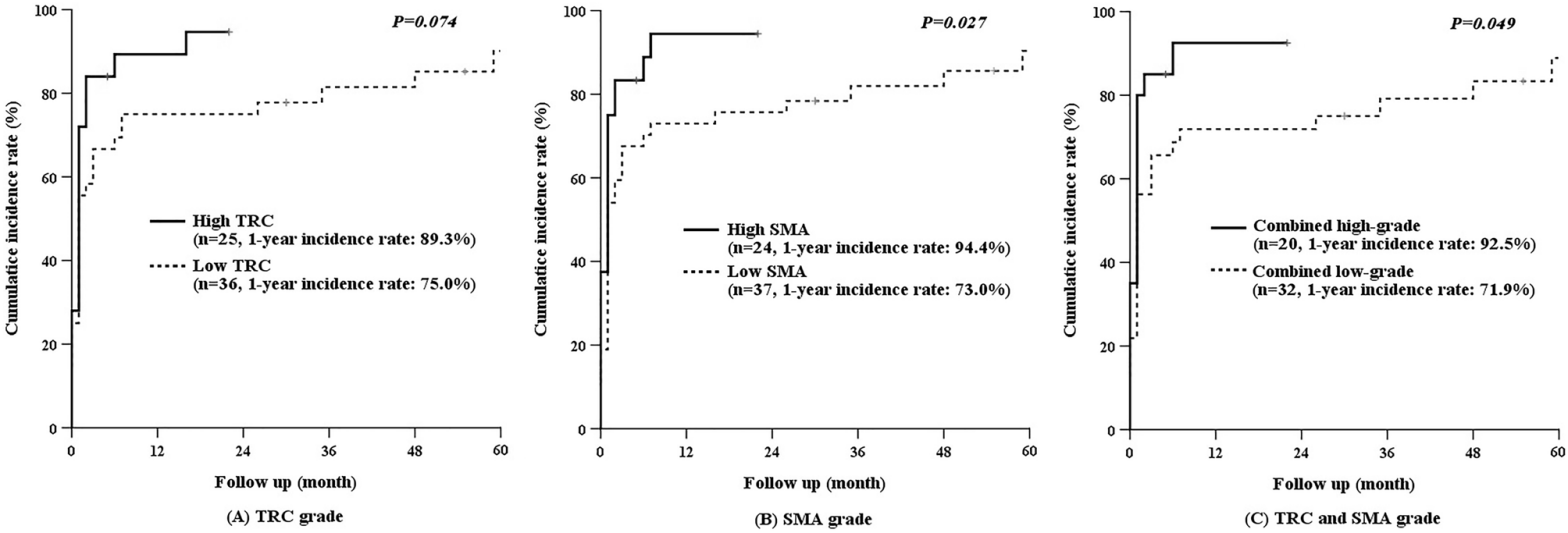
Cumulative incidence of hyperglycemia and pancreoprivic diabetes TRC and SMA staining. Hyperglycemia/pancreoprivic diabetes developed earlier and more commonly in patients with high-grade TRC (**A**) and SMA (**B**) staining. The cumulative incidence of hyperglycemia/pancreoprivic diabetes was higher in the patients with combined high-grade TRC and SMA staining than in the patients with combined low-grade staining (**C**).

Based on the FBG test results, hyperglycemia and pancreoprivic diabetes were diagnosed in 54 (88.5%) patients during the follow-up period. The average interval between surgery and the diagnosis of hyperglycemia or pancreoprivic diabetes was 4.4 months. When analyzed according to the severity of fibrosis, the high-grade TRC group developed hyperglycemia and pancreoprivic diabetes earlier after surgery and more often than the low-grade TRC group (Fig. 3A: 1-year cumulative incidence, 89.3% vs. 75%, *P* = 0.074). Hyperglycemia and pancreoprivic diabetes also occurred earlier and more commonly in the high-grade SMA group than in the low-grade SMA group (Fig. 3B: 1-year cumulative incidence, 94.4% vs. 73%, *P* = 0.027). The cumulative incidence of hyperglycemia and pancreoprivic diabetes in the combined high-grade groups was higher than in the combined low-grade groups (Fig. 3C, 1-year cumulative incidence, 92.5% vs. 71.9%, *P* = 0.049).

**Figure 4:**
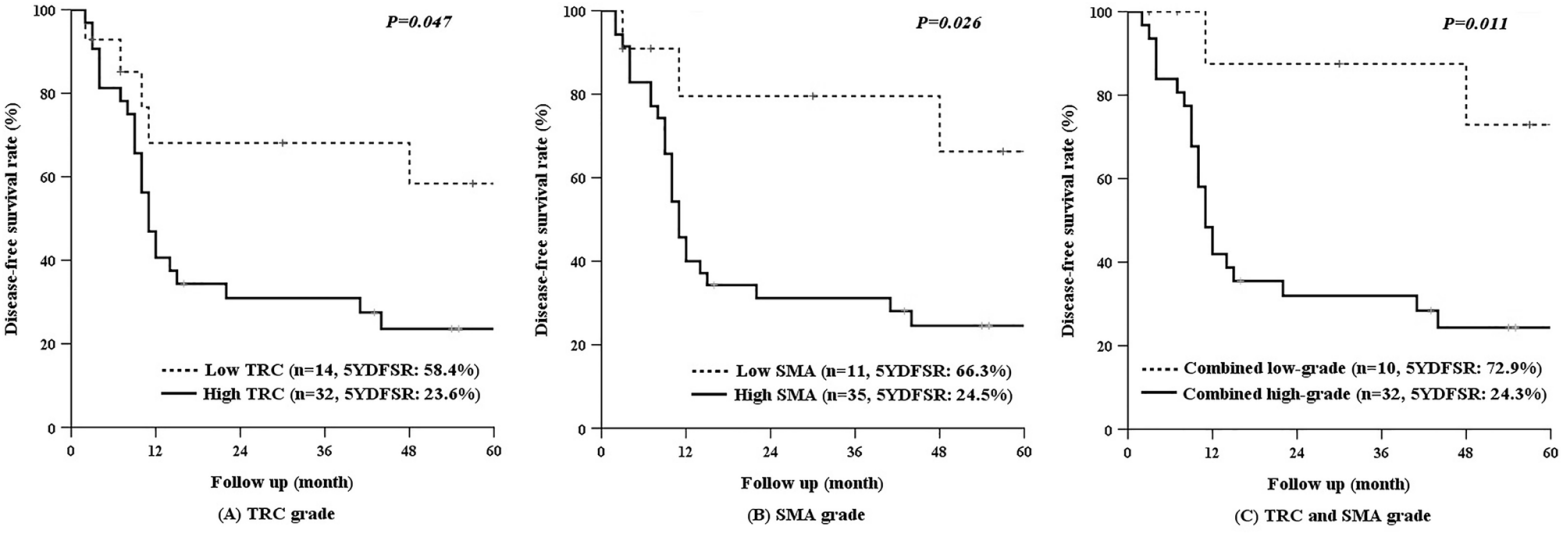
Disease-free survival of patients with pancreatic cancer by TRC and SMA grades. The 5-year disease-free survival was much lower in the patients with high-grade TRC (**A**) or SMA (**B**) staining. Disease-free survival was significantly lower in the patients with combined high-grade TRC and SMA staining than in patients in the combined low-grade group (**C**).

### Disease-free survival of pancreatic cancer by PF grade

The disease-free survival of patients with pancreatic cancer was analyzed according to the grade of PF. Among 46 patients, 36 and 10 with pancreatic ductal adenocarcinoma and invasive IPMN, respectively, were included in the analysis. The 5-year disease-free survival of the high-grade TRC group was worse than that of the low-grade TRC group (Fig.4A, 23.6% vs. 58.4%, *P* = 0.047). The 5-year disease-free survival of the high-grade SMA group was also worse than that of the low-grade SMA group (Fig. 4B, 24.5% vs. 66.3%, *P* = 0.026). When patients were stratified by grade alone, the combined high-grade group demonstrated worse 5-year disease-free survival than the combined low-grade group with the greatest predictive value (Fig. 4C, 24.3% vs. 72.9%, *P* = 0.011).

## Discussion

PSCs are myofibroblast-like cells in the pancreas that play an important role in fibrogenesis^17^. PSCs are activated from their quiescent states in chronic pancreatitis and pancreatic cancer. Initial studies localized PSCs to the interlobular and interacinar regions of the pancreas but not to the islets of Langerhans^18,19^; however, recent models have identified activated PSCs in the islets of animal models^20^. Pancreatic islet fibrosis is often observed in patients with diabetes mellitus^21,22^. In 2017, Lee et al. reported greater extents of islet fibrosis in patients with type 2 diabetes compared to patients without diabetes (15% vs. 10.3%, *P* = 0.05)^23^. We hypothesized that PF at the time of surgery has an impact on the development of pancreoprivic diabetes after PD.

Our study utilized the degree of SMA and TRC staining in representative specimen sections to evaluate the severity of PF. When fibrogenesis is activated, PSCs express α-SMA and produce extracellular matrix proteins^24^. As fibrogenesis progresses, fibrous tissue becomes denser and is composed of keratin, muscle fibers, and collagen^25^. TRC stains the collagen component of the tissue. In our study, the SMA and TRC gradings were utilized to reflect PSC activity during the early and advanced stages of fibrosis, respectively. Patients with high-grade TRC and SMA staining have advanced and actively progressing fibrosis.

In 2006, Tran et al*.* evaluated the correlation between PF and pancreatic insufficiency after PD^26^. The extent of fibrosis, remaining functional exocrine tissue, and endocrine tissue loss were assessed on the plane of the pancreatic resection margin by an experienced gastrointestinal pathologist. The study reported that the extent of exocrine insufficiency and loss of endocrine tissue were strongly correlated with PF; however, neither PF nor endocrine tissue loss were correlated with the development of pancreoprivic diabetes. Our study investigated the effect of PF at the time of surgery on the onset of hyperglycemia or pancreoprivic diabetes after surgery. Patients with higher FBG levels after surgery demonstrated higher grades of TRC and SMA staining. In addition, the cumulative incidence of hyperglycemia and pancreoprivic diabetes was higher in the groups with high-grade TRC and SMA staining. Therefore, the degree of PF might be considered as a predictive factor for the development of pancreoprivic diabetes in patients who undergo PD.

An analysis of high-grade TRC and SMA staining in the combined high-grade group demonstrated significant differences compared to the combined low-grade group. First, most patients (90%) in the combined high-grade group had primary pancreatic disease. Primary pancreatic disease, such as pancreatic ductal carcinoma, is characterized by severe fibrosis because desmoplasia, which is a component of these diseases, results in the production of densely fibrotic stroma^27^. Pancreatic duct obstruction and pancreatitis after endoscopic retrograde cholangiopancreatography also contribute to fibrogenesis in pancreatic disease. Second, the combined low-grade group demonstrated higher BMIs than the combined high-grade group. This result could be influenced by an increased proportion of pancreatic fat in patients with high BMI. Saisho et al. reported that the fat/parenchyma ratio was increased in overweight and obese patients compared to lean patients^28^. However, Gaujoux et al. reported that PF was not correlated with BMI or fatty pancreas^29^. In addition, Matsuda et al. reported that fatty change of the pancreas seems to be related to subsequent PF^30^. Therefore, further research is needed to identify the relationship between BMI and PF.

In 2018, Scholten et al*.* conducted a systematic review and meta-analysis regarding pancreoprivic diabetes after PD^4^. The study included 1,121 patients from 22 studies. The incidence of pancreoprivic diabetes after PD in the study was 16% (95% confidence interval, 12–20%). Our study examined 61 patients without preoperative diabetes and documented hyperglycemia in 54 (88.5%) patients after PD. Among these patients, 40 (65.6%) developed pancreoprivic diabetes. Our results demonstrated a higher incidence of pancreoprivic diabetes compared to other studies. Our results may be influenced by the small sample size and the high proportion of primary pancreatic disease in our study population. The patients in our study also underwent a strict follow-up protocol that required a FBG test every 3 months.

Among patients with pancreatic cancer, there was a significant difference in the disease-free survival depending on the degree of fibrosis. The 5-year disease-free survival of patients with high-grade TRC and SMA staining was much lower than that of patients with low-grade staining. These results may be due to the chemoresistance of dense fibrotic stroma. When PSCs are activated, fibrosis progresses through excessive extracellular matrix deposition, which compresses and distorts intratumoral vasculature and results in hypoxia^31^. Hypoxia stimulates the epithelial-mesenchymal transition of cancer cells, which are more chemoresistant. Fibrosis also sequesters chemotherapeutics in the stromal compartment, which impairs successful drug delivery to the cancer cells.

The present study has some limitations. First, the study had a retrospective design and involved a small sample of patients in a single institution. However, we prospectively collected clinical data by evaluating FBG levels on an outpatient basis at 3-month intervals. Second, we evaluated the grade of PF at the time of surgery, which did not reflect the severity of PF at the time when pancreoprivic diabetes was diagnosed. The TRC and SMA grading utilized in our study was an indirect inference of the postoperative PF status of our patients. Third, there were differences between the combined high-grade group and the combined low-grade group for disease entity and adjuvant chemotherapy. The heterogeneity between the two groups imposed limitations for evaluating the effect of PF on the development of pancreoprivic diabetes. Lastly, our study did not consider the effects of lifestyle, such as diet and exercise, on the development of type 3 diabetes. However, to the best of our knowledge, this is the first study that analyzed the relationship between PF and the development of pancreoprivic diabetes.

In conclusion, hyperglycemia and pancreoprivic diabetes after PD were more likely to occur in patients with severe PF. PF in pancreatic cancer affects disease-free survival. Assessing PF on a histopathologic level may help predict patient prognosis after PD. Further research with larger patient samples and longer follow-up times should be conducted.

## Supplementary Information

Supplementary Information.

## Data Availability

All data used during the current study are included in this published article or are available from the corresponding author upon reasonable request*.*
