# Supplementary material for: Association between pancreatic fibrosis and development of pancreoprivic diabetes after pancreaticoduodenectomy
Source: Sci Rep. 2021 Dec 7;11:23538. doi: 10.1038/s41598-021-02858-z (PMC8651673; doi:10.1038/s41598-021-02858-z)
Supplement: Supplementary file 1 — Supplementary Information. [file 41598_2021_2858_MOESM1_ESM.docx]

**Supplementary table 1. Medication of patients with preoperative diabetes**

| **Medication** | **Total**  **(N=34)** |
| --- | --- |
| **Biguanide** | 2 (5.9%) |
| **Biguanide + Sulfonylurea** | 5 (14.7%) |
| **Biguanide + Thiazolidinedione** | 4 (11.8%) |
| **Biguanide + DPP4 inhibitor** | 6 (17.7%) |
| **Biguanide + Sulfonylurea + DPP4 inhibitor** | 2 (5.9%) |
| **Biguanide + Sulfonylurea + DPP4 inhibitor + Thiazolidinedione** | 1 (2.9%) |
| **Sulfonylurea** | 2 (5.9%) |
| **Sulfonylurea + Thiazolidinedione** | 1 (2.9%) |
| **Long-acting insulin + Biguanide + DPP4 inhibitor** | 2 (5.9%) |
| **Long-acting insulin + short-acting insulin** | 1 (2.9%) |
| **Diet and life style modification** | 8 (23.5%) |

Abbreviation: DPP4, dipeptidyl peptidase-4

**Supplementary table 2. Oral glucose tolerance test results of patients with pancreoprivic diabetes**

| **OGTT 2-hour PG** | **Total**  **(N=40)** |
| --- | --- |
| **2-h PG > 200 mg/dl** | 14 (35.0%) |
| **200 mg/dl ≥ 2-h PG > 150 mg/dl** | 15 (37.5%) |
| **150 mg/dl ≥ 2-h PG** | 11 (27.5%) |

Abbreviation: OGTT, oral glucose tolerance test; PG, plasma glucose
